# The Neural Substrates of Social Influence on Decision Making

**DOI:** 10.1371/journal.pone.0052630

**Published:** 2013-01-09

**Authors:** Damon Tomlin, Andrea Nedic, Deborah A. Prentice, Philip Holmes, Jonathan D. Cohen

**Affiliations:** 1 Princeton Neuroscience Institute, Princeton University, Princeton, New Jersey, United States of America; 2 Department of Electrical Engineering, Princeton University, Princeton, New Jersey, United States of America; 3 Department of Psychology, Princeton University, Princeton, New Jersey, United States of America; 4 Department of Mechanical and Aerospace Engineering, Princeton University, Princeton, New Jersey, United States of America; 5 Program in Applied and Computational Mathematics, Princeton University, Princeton, New Jersey, United States of America; University of Minnesota, United States of America

## Abstract

The mechanisms that govern human learning and decision making under uncertainty have been the focus of intense behavioral and, more recently, neuroscientific investigation. Substantial progress has been made in building models of the processes involved, and identifying underlying neural mechanisms using simple, two-alternative forced choice decision tasks. However, less attention has been given to how social information influences these processes, and the neural systems that mediate this influence. Here we sought to address these questions by using tasks similar to ones that have been used to study individual decision making behavior, and adding conditions in which participants were given trial-by-trial information about the performance of other individuals (their choices and/or their rewards) simultaneously playing the same tasks. We asked two questions: How does such information about the behavior of others influence performance in otherwise simple decision tasks, and what neural systems mediate this influence? We found that bilateral insula exhibited a parametric relationship to the degree of misalignment of the individual's performance with those of others in the group. Furthermore, activity in the bilateral insula significantly predicted participants' subsequent choices to align their behavior with others in the group when they were misaligned either in their choices (independent of success) or their degree of success (independent of specific choices). These findings add to the growing body of empirical data suggesting that the insula participates in an important way in social information processing and decision making.

## Introduction

The mechanisms that govern human learning and decision making under uncertainty have been the focus of intense behavioral and, more recently, neuroscientific investigation. An important focus has been on performance in two alternative forced choice (TAFC) decision tasks, in which the two choices are associated with different probabilities of reward, and the participant must discover how to maximize their reward by sampling each option 1. Models based on simple principles of reinforcement learning and information integration have begun to reveal the processes responsible for the performance of individuals in such tasks 2–5. However, relatively little is known about how social information — that is, the experience of other individuals engaged in the same task — influences these decision making processes, and even less is known about the neural mechanisms that mediate these influences.

Recently, neuroimaging has been used to identify what neural systems are responsive to the presence and use of social information. However, these have tended either to focus on the responses to social factors rather than how these factors contribute to decision making 6,7, or to examine decisions in the domain of preferences, which lack an objective measure over which their merits can be compared 8–12. Those studies that *have* employed objective outcomes have used social agents with: 1) roles significantly different from the participant's, thereby precluding the comparison of decisions 13–18, or 2) fictitious behavior designed to balance the experimental design rather than mimic actual human decisions 19,20.

In the present study, our goal was to use the simplest and most direct design possible to address a particular gap we perceived to be present in this area of research: the use of simple TAFC tasks, in which individual performance has been well-characterized, to study how the introduction of specific forms of social information under tightly controlled conditions influences decision making, and identify the neural systems involved. Our motivation for this approach was two-fold: 1) to generate data from tasks that have been subjected previously to computational modeling, so that these models can be extended to address the influence of social information on decision making; and 2) to acquire neural data that could identify brain systems responsive to, and involved in mediating the influence of, social information. Results pertaining to the first objective are the subject of separate reports 21–23. Here, we focus on results related to the second objective.

## Materials And Methods

### Participants

Participants were recruited at Baylor College of Medicine via email and word of mouth, and informed consent was obtained according to protocols approved by Baylor College of Medicine and Princeton University's Institutional Review Boards. Groups of five participants engaged in a series of decision making tasks while functional magnetic resonance imaging (fMRI) data were acquired 24. Participants did not meet prior to the experiment, nor did they see one another afterward. On a few occasions, the initiation of synchronized data acquisition failed on one or two scanners. While this did not interfere with the collection of behavioral data (and was not apparent to those participants whose scanners *did* acquire data), the imaging data for the affected participants were lost. As noted below, there were four social conditions crossed with the six tasks, yielding a 6×4 design. Groups were collected until a minimum of 15 individual fMRI data sets filled each of the 24 cells of the design (after excluding for excessive head motion). This yielded a behavioral set of 23 groups (n = 115 individuals; 68 female, 47 male; ages 18–57, with a mean age of 29), and an imaging subset of 86 participants (n = 86 individuals; 52 female, 34 male).

### Decision Making Tasks In A Social Context

Groups of five participants each played a set of six simple decision making games involving a series of two-alternative forced choices. Visual feedback was presented after each trial on a rear projection screen and was viewed by participants via a mirror on the acquisition coil. Behavioral responses were recorded by an optical button box placed in the right hand.

The tasks used in the games were similar to ones used previously for studying individual decision making behavior 1,3,25. For each trial in each task, each participant chose between two buttons, “A” and “B,” that then produced a reward. The reward was calculated using a deterministic reward function based on the participant's choice history (percentage of the last twenty choices allocated to button A). This reward was then shown to the participant prior to the next choice. This paradigm allowed participants' earnings to change over time as they either continued to press a given button, or chose the other one. Participants were not instructed that the history of their choices was a key determinant of the reward earned, and none reported being aware of this dependency. The six tasks differed according to the reward function used (these are described in the Supporting Information, and in-depth behavioral and computational analyses of these have been reported elsewhere 21–23). The effect of social information upon brain activity and subsequent choices – the focus of the present report – was comparable across the tasks. Thus, for present purposes, we combine data across tasks.

**Figure 1:**
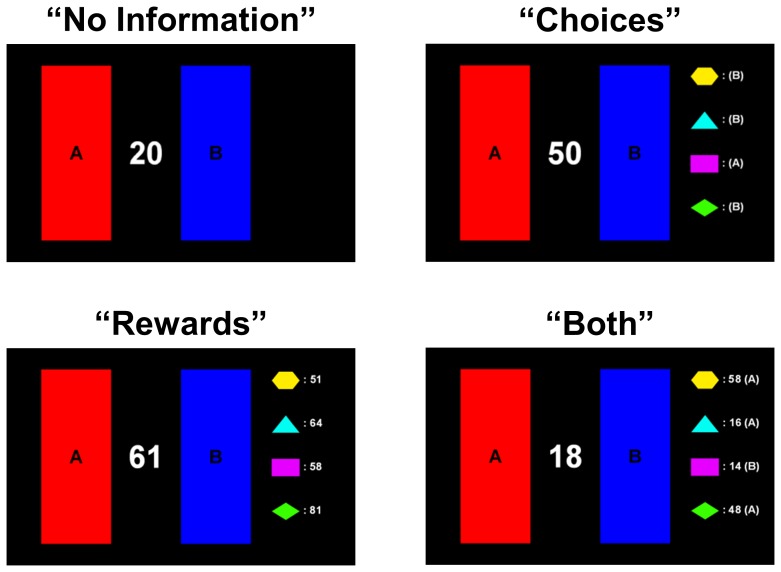
Social conditions within the multi-person decision making tasks. Participants played each task under a social condition that determined what information was available about other group members. In the No Information condition, participants played a task without social information. In the Choices condition, the button previously chosen by each other group member was shown, and was updated for each decision. In the Rewards condition, the number of points earned by each other group member was shown, and was updated synchronously as in the Choices condition. Finally, the Both condition displayed both Choices and Rewards information on each trial.

Each of the five participants in a group made a series of 150 decisions in each of the six games. Participants' choices were synchronized across the group, and all group members performed the same task in each game. Thus, information about other group members could be informative, although the reward functions for each participant was always independent of the others (no competition), and participants could not communicate directly (no cooperation). While information regarding their own most recent reward was always available to participants, information about the other group members varied from task to task. Each game was played in one of four social conditions (Figure 1). In the “No Information” condition nothing was displayed about the other participants. In the “Choices” condition, participants were shown which buttons each of the other four participants had pressed in the last trial, but not their rewards. In the “Rewards” condition, participants were shown how many points each of the other participants earned on the last trial, but not their choices. Finally, in the “Both” condition, participants were shown both the buttons pressed *and* how many points were earned by each other group member. Information about other players was displayed beside a symbol corresponding to that player which remained consistent across the entire experiment. These symbols were not shown during the No Information condition.

Participants were informed that the other participants were playing the same game, but that the choices and earnings of the other group members would not affect their own earnings, and that the purpose of the shared social information was only to allow group members to observe one another. This incentive structure was selected because it allowed for the provision of social information, but precluded the complicating influence of either competitive or cooperative group behaviors.

Each group played six games, each of which involved a single task paired with a single social condition. Every group played each task and experienced each social condition at least once. Assignment of tasks to social conditions was counterbalanced across groups using a Latin-Square design. Consecutive games always involved different tasks and were played under different social conditions. For example, a group might play one task in the Choices condition as the first game, a different task in the No Information condition as the second game, yet another task in the Rewards condition as the third game, and so on.

The earnings during each game were assigned a random scale factor between 50 and 99 to prevent participants from knowing if they had obtained the maximum possible reward within a given task at any time. The number of points accrued, which determined participants' compensation ($30 to $50 USD), was calculated after normalizing by this scale factor so that each task had the same potential payoff. Participants were informed of these compensation procedures prior to the experiment.

The decisions in each trial had a deadline of 1.7 seconds. For decisions not made in time, the computer used the button chosen on the previous trial as the participant's current choice. Decisions or the passage of the deadline were confirmed to participants by the “A” and “B” buttons turning gray. This state persisted until the trial length was 2.5 seconds, yielding a minimum inter-trial interval of .8 seconds and synchronizing participants' decisions. After this period, the buttons regained their colors and the relevant reward and social information for the previous trial were shown. Tasks were separated by a screen lasting eight seconds and indicating the social condition in which the next task would be performed.

### Data Acquisition And Preprocessing

Functional imaging data 26,27 were collected using two Siemens 3.0 Tesla Allegra scanners and three Siemens 3.0 Tesla Trio scanners (combinations of task and social condition were balanced across scanners). Each session included a high resolution, T1-weighted scan (MP-RAGE; Siemens). Whole-brain imaging was collected during the tasks in a single session using echo-planar imaging with a repetition time (TR) of 2000 ms, echo time (TE) of 40 ms, and a flip angle of 90°. The images were acquired as matrices of 64×64×26 voxels aligned to the anterior and posterior commissures of the corpus callosum, resulting in voxels with a resolution of 3.4×3.4×4.0 mm.

Image preprocessing was performed using SPM8 28,29. Slice timing correction was followed by realignment to the first functional scan using a six-parameter rigid-body transformation. The mean of the realigned images was coregistered to the T1-weighted structural image using a twelve-parameter affine transformation. Tissue segmentation was determined for each structural image, and the gray matter designated by this classification was used for spatial normalization by applying a twelve-parameter affine transformation. The functional images were then normalized and smoothed with an 8 mm FWHM Gaussian kernel for inter-subject analyses.

### Data Analysis

Our approach to analysis was designed to meet two goals: 1) identify brain areas responsive to social information; and 2) determine the extent to which social information and/or corresponding brain activity was predictive of subsequent behavior. Toward these ends, we conducted two types of analyses, one using standard linear regression and a second using logistic regression.

#### Glm Analysis: Identification Of Brain Areas Responsive To Social Information

We used SPM8 29,30 to implement a standard general linear model (GLM) for each participant, that included regressor representing timing of decisions and nature of the social information on each trial, as well as variables of no interest (head motion, and the effects of absolute reward – i.e., independent of social information). The regressor for head motion was constructed from the motion values computed by the 6-parameter rigid body transformation used for spatial realignment. To control for the effects of absolute reward, regressors were included that coded the reward amplitude on each trial and its derivative (i.e., its magnitude relative to the previous trial). For the effects of social information, a separate regressor was constructed for each social condition, as follows: First, a base regressor was constructed from a series of delta functions placed at each time point at which the participant submitted a decision (due to the timing of the task, delta functions placed at the screen onsets produced the same qualitative result); for trials in which participants did not respond before the deadline, delta functions were placed at the deadline. Next, the delta function corresponding to each decision was scaled by the social information available to the participant during that decision (quantified as described below). Finally, the scaled delta functions were convolved with a hemodynamic response function (modeled via two gamma functions) to simulate the time course of the BOLD response.

**Figure 2:**
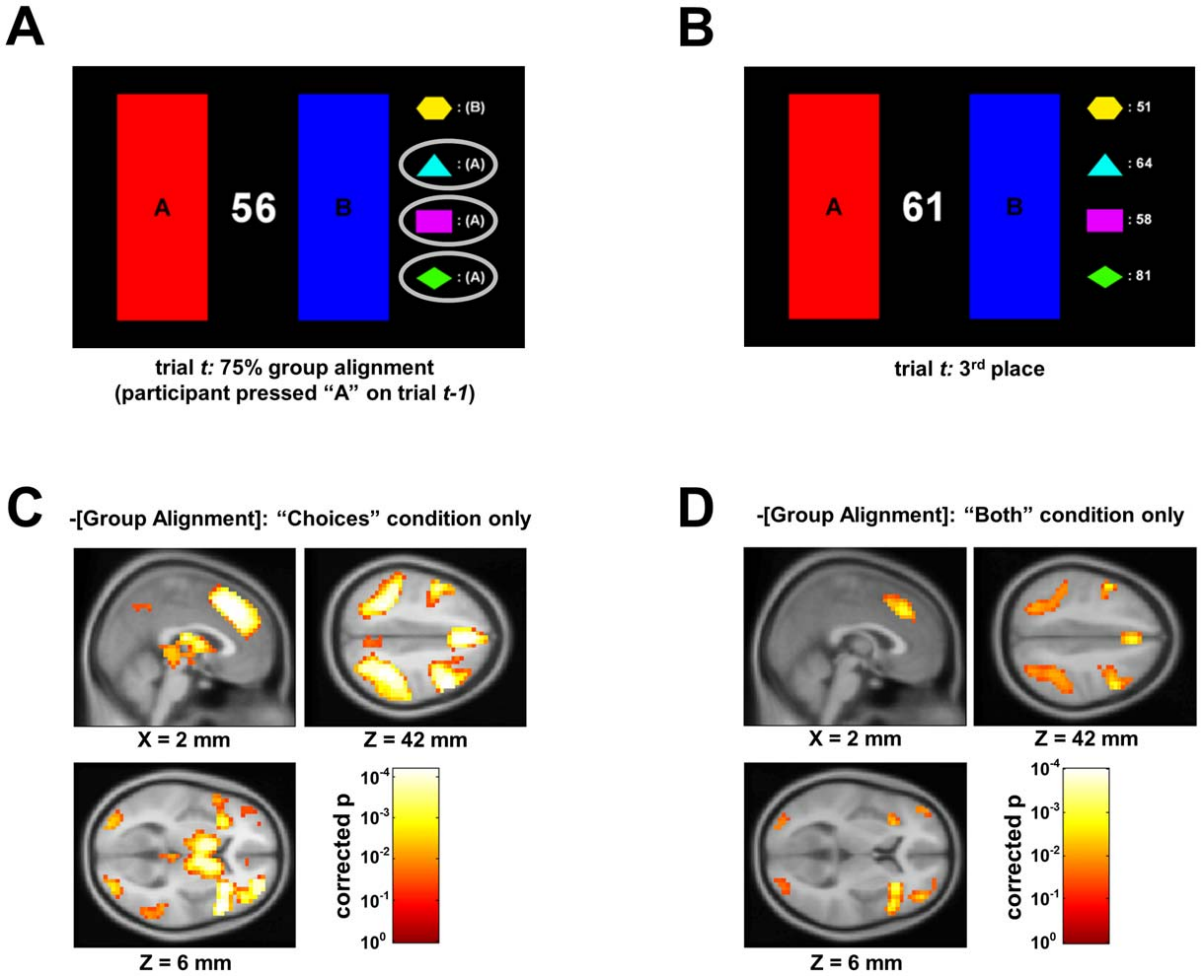
BOLD activity is correlated with continuous measures of marginality. (A) The group alignment metric. A participant's group alignment metric was defined as the percentage of other group members who made the same choice during the previous trial. Information regarding the buttons pressed by each group member in the previous trial was revealed at the onset of the current trial. (B) The reward rank metric. A participant's reward rank metric was defined as the ordinal value of the participant's earnings relative to those of the other participants on each trial, with higher values indicating greater relative rewards. Information regarding the earnings of each group member in the previous trial was revealed at the onset of the current trial. (C) Effect of group alignment in the Choices condition. In the Choices condition, activity in the insula, thalamus, DLPFC, dACC, and parietal cortex was inversely proportional to the participant's group alignment (p<.05 corrected; see Table 1). (D) Effect of group alignment in the Both condition. In the Both condition, activity in the insula, DLPFC, dACC, and parietal cortex was inversely proportional to the participant's group alignment (p<.05 corrected; see Table 2).

To quantify social information on a trial-by-trial basis, we defined two metrics: one for choices (“group alignment”) and another for rewards (“reward rank”). Group alignment quantified the similarity of each participant's choice to those of other group members during the Choices and Both conditions (Figure 2A). For a given participant and choice, the metric was defined as the proportion of the group that chose the same button as the participant on the previous trial (excluding the participant in question). The metric varied between 0 and 1 in increments of .25, with 1 indicating a unanimous decision and 0 indicating that all other members had made the opposite choice. To quantify social information about reward, a reward rank metric was calculated as the ordinal value of the participant's earnings relative to those of the other participants on each trial, with higher values indicating higher rank and values between whole numbers representing ties (Figure 2B). Those metrics based on information unavailable during a given condition (e.g., information regarding others' choices in the Rewards condition) were excluded.

The GLM for each participant was fit voxel-wise to the BOLD data for that participant, and a random effects analysis was performed across participants for the betas computed for each regressor. Data were then cluster-thresholded, via a nonparametric permutation test (Threshold-Free Cluster Enhancement, 31–33), to identify regions of interest (ROIs) that surpassed a level of statistical significance of p<.05.

#### Logistic Regression Model: Effects Of Social Information And Brain Activity On Behavior

The second goal of our analyses was to examine how social information and corresponding brain activity influenced subsequent choice behavior. To do so, we used logistic regression to test the extent to which social information and/or brain activity in each ROI predicted “switching” from one button to the other on each trial. We chose switching (rather than specific button choices) as it indicated a shift in a participant's preference over the buttons, and was therefore likely to be most sensitive to the impact of social information and/or related brain activity on decision making behavior. Because button switching is a binary variable, it required the use of logistic (rather than linear) regression. The regression was carried out separately for each of the ROIs identified in the GLM described above, and estimated the probability of switching using a weighted function of: a) the social information available to the participant on each trial (i.e., group alignment and/or reward rank); and b) brain activity (the BOLD response) for the given ROI.

The model was fit by maximizing its ability to predict switching behavior on each trial (excluding the first choice), defined by a binary variable: whether or not the button pressed by the participant was the same as that on the previous choice (1 for switches, 0 for non-switches). The probability of switching on trial *i* was defined by:
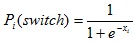
where the variable 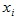 was defined by the following sum:
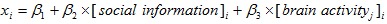
In which *[social information]_i_* designated the quantified value of social information (group alignment or reward rank, depending upon social condition) available on trial *i*, and *[brain activity_j_]_i_* designated the mean BOLD response for ROI *j* on trial *i*. For each ROI, three regressions were carred out, implementing models of increasing complexity. The first sought to explain behavior by fitting only the parameter β_1_ (i.e., by setting β_2_ and β_3_ to zero), capturing the participant's bias toward (average probability of) switching regardless of social information or brain activity. The second model added social information (that was scaled to range from 0 to 1) by fitting both β_1_ and β_2_. The third model was hierarchical: first we fit parameters β_1_ and β_2_ to account for the effects of the participant's bias and of social information on switching; we then fit the remaining variance (residuals) by estimating β_3_, reflecting the effects of brain activity on switching behavior independent of social information. This latter regression was carried out since social information and the BOLD signals were known to be correlated (given the way in which the regions of interest were defined); using hierarchical regression allowed us to determine the extent to which neural activity explained switching behavior above and beyond the effects of social information. For this hierarchical regression, brain activity in each region of interest was computed by extracting the BOLD signal during the decision epoch of each trial (shifted by four seconds to account for hemodynamic lag), averaging across voxels in that region of interest, and z-scoring this mean across trials. Because the Both condition involved combining Choices and Rewards feedback, separate regressors were included for the metric associated with each type of social information (group alignment and reward rank):
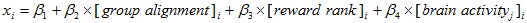



A final model was created to estimate any statistical interaction between social information and the BOLD signal from the regions of interest. However, these interactions were not significant for any of the signals tested, and thus are not considered further here. Parameter values were estimated using log-likelihood maximization via MATLAB's function minimization routine 34. Participants exhibiting less than three instances of switching (6% of the total sample) were excluded from the analysis.

Because the models that included brain activity contained one more parameter than those which used only the baseline rate of switching and social information, we used Akaike's Information Criterion (AIC) to assess whether predictive power was significantly improved by addition of the brain activity parameter. We then conducted paired t-tests to determine whether the brain activity in each region significantly increased these AIC values (i.e., significantly predicted behavior) across participants.

## Results

### Brain Regions Sensitive To Social Information

**Figure 3:**
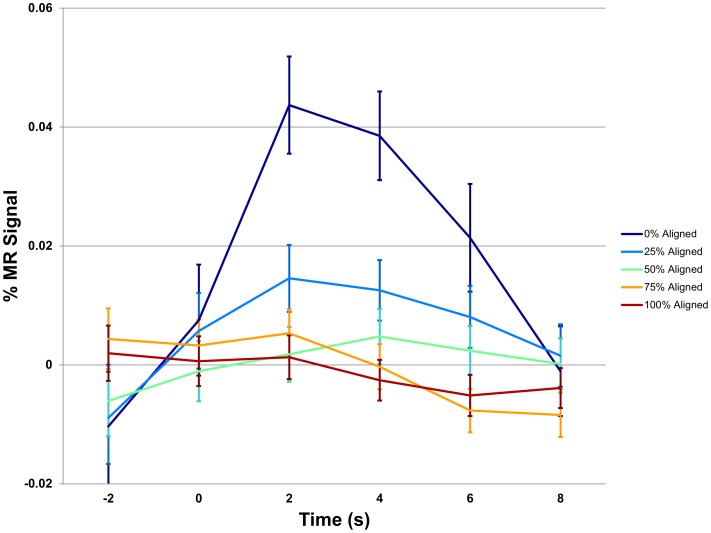
Bilateral insula exhibits graded response to group alignment. After performing the statistical tests described in Figure 2, we examined the time course of the MR signal exhibited by the bilateral insula in the Choices condition. Time courses corresponding to each decision were categorized according to their group alignment values and plotted for the five levels of alignment. The average BOLD signal was highest for choices for which group alignment was lowest (ANOVA at t+4 seconds: p<10^−4^). Error bars represent standard error of the mean (SEM). Similar effects were observed for the other regions shown in Figure 2C (note that because participants' decisions were not systematically in register with the onset of image acquisition, linear interpolation was used to align the time-courses of the BOLD signal across decisions for averaging).

For each participant included in the GLM analysis (n = 86 individuals; 52 female, 34 male), group alignment in the Choices condition was correlated negatively with activity in several regions (Figure 2C). That is, as participants' group alignment decreased, and therefore their marginality relative to the group increased, so did activity in these areas. The regions identified were the insula, dorsal anterior cingulate cortex (dACC), dorsolateral prefrontal cortex (DLPFC), parietal cortex, medial thalamus (with portions of this activation overlapping with the dorsal caudate), all bilateral (p<.05 corrected; see Table 1). When data from the Both condition were analyzed using the same method, a similar network of regions (with the exception of the thalamus) was shown to be active, while no additional regions showed a correlation (Figure 2D; see Table 2). Analysis of time courses of activity in these regions confirmed that the BOLD response increased as fewer group members made the same choice as the participant (ANOVA at t+4 seconds: for the insula, p<10^−4^; see Figure 3). GLM analysis of the Rewards condition revealed no regions in which activity varied parametrically with reward rank. However, our findings in the Choices condition led us to hypothesize that the regions shown in Figure 2C exhibited activity correlated with reward rank, and that such an effect was not apparent in the GLM due to the larger number of values that the reward rank metric could attain (thereby reducing the amount of data corresponding to each possible value). Post-hoc analysis of the Rewards condition, using masks based on the regions shown in Figure 2C, demonstrated that reward rank *was* negatively correlated with insula activity at t+4 seconds (p<.01, fixed effects analysis corrected for multiple comparisons). Similarly, GLM analysis of the Both condition revealed no regions that varied with reward rank. However, post-hoc analysis again revealed that reward rank was negatively correlated with insula activity at t+4 seconds (p<.01, fixed effects analysis corrected for multiple comparisons).

**Table 1:** Activations for group alignment analysis in the Choices condition.

Brain Region	MNI Coordinates	p value	Voxels
R Insula	(30, 16, −14)	p<10^−4^	279
L Insula	(−30, 20, −10)	p<10^−3^	168
R Thalamus	(10, 0, 10)	p<10^−4^	340
L Thalamus	(−6, 0, 2)	p<10^−3^	267
R DLPFC	(54, 8, 14)	p<10^−4^	1009
L DLPFC	(−42, 0, 26)	p<10^−3^	480
dACC	(6, 36, 30)	p<10^−4^	399
R Parietal	(34, −48, 38)	p<10^−4^	1055
L Parietal	(−30, −52, 42)	p<10^−4^	565

Activations are shown for regions surpassing a statistical threshold of p<.05 (corrected for multiple comparisons using cluster-mass correction). L, left hemisphere; R, right hemisphere. Coordinates and statistical values are shown for the voxel of highest significance within each cluster.

doi:10.1371/journal.pone.0052630.t001

**Table 2:** Activations for group alignment analysis in the Both condition.

Brain Region	MNI Coordinates	p value	Voxels
R Insula	(34, 24, −10)	p<10^−4^	170
L Insula	(−30, 24, −2)	p<.005	70
R DLPFC	(54, 12, 14)	p<10^−4^	519
L DLPFC	(−42, 4, 30)	p<10^−4^	264
dACC	(2, 32, 38)	p<.005	78
R Parietal	(38, −52, 46)	p<.01	212
L Parietal	(−26, −72, 30)	p<.01	195

Activations are shown for regions surpassing a statistical threshold of p<.05 (corrected for multiple comparisons using cluster-mass correction). L, left hemisphere; R, right hemisphere. Coordinates and statistical values are shown for the voxel of highest significance within each cluster.

doi:10.1371/journal.pone.0052630.t002

### Influence Of Social Information On Brain Activity And Switching Behavior

As described above, the GLM analysis revealed several regions in which activity was significantly correlated with social information. We used hierarchical logistic regression to identify which of these regions significantly predicted switching behavior above and beyond the information provided in each social condition. Because activity was most robust in the Choices condition, we used the regions identified in that condition (see Figure 2C) for the hierarchical regressions. This also allowed us to keep the regions of interest for these analyses consistent across the social conditions.

**Figure 4:**
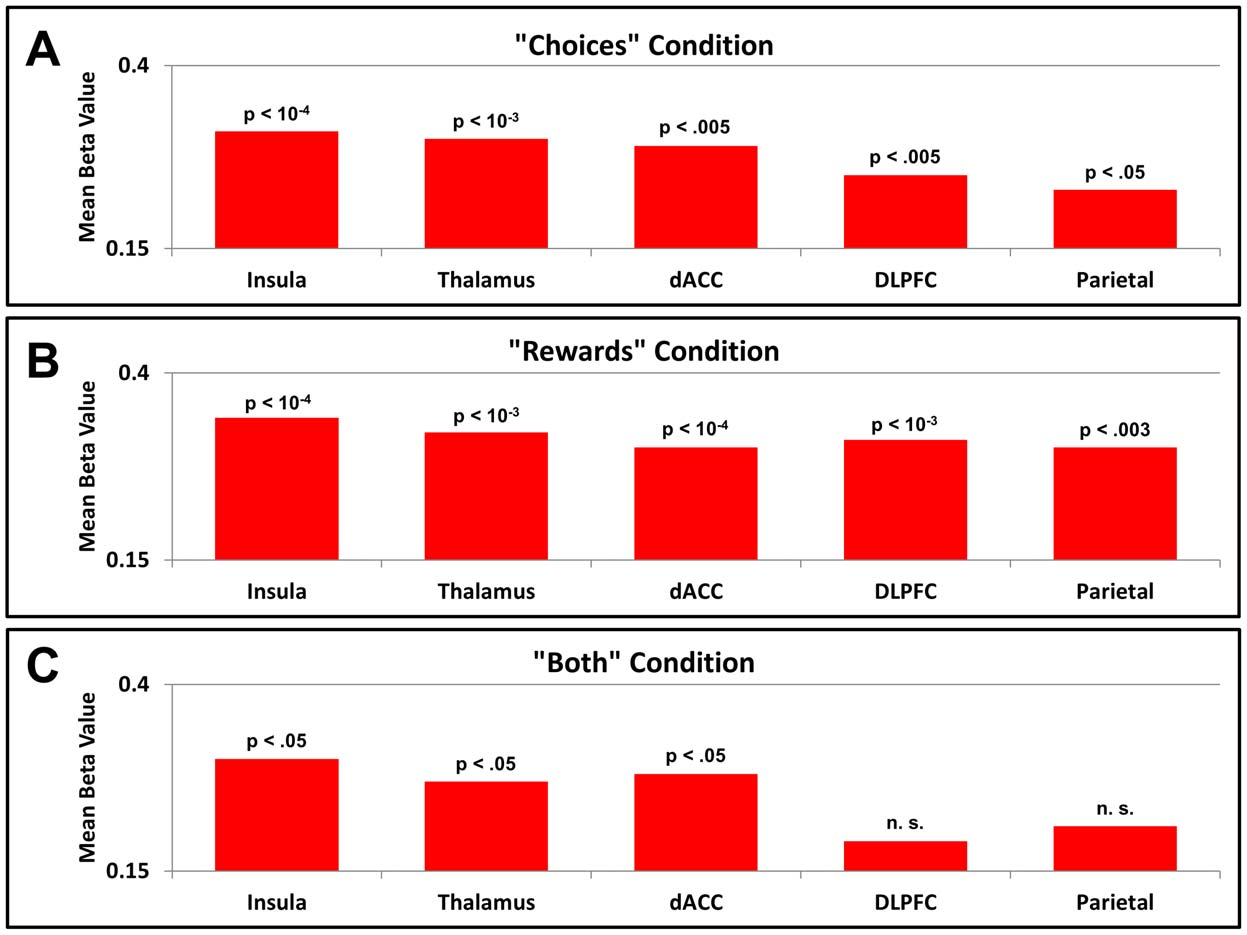
Comparison of explanatory power across regions. Using hierarchical logistic regression, beta values were fitted to BOLD signals that accounted for the most variance in each participant's button switching behavior. These beta values were calculated for each of the regions shown in Figure 2C, and the means of these beta values are shown for each region and social condition. The values for Akaike's Information Criterion obtained when incorporating these BOLD signals were compared to those obtained when including behavioral metrics, and the results of these statistical tests are shown above each beta (paired t tests corrected for multiple comparisons). (A) Beta values for the Choices condition. Although BOLD signals from all regions shown in Figure 2C produced significantly better fits than those using behavioral metrics alone, the insula accounted for the most variance. (B) Beta values for the Rewards condition. As in the Choices condition, all regions tested produced significantly better fits than those using behavioral metrics only, with the insula again outperforming the other regions. (C) Beta values for the Both condition. In this case, only the insula, thalamus, and dACC produced significantly better fits than those using behavioral metrics alone, with the insula once again accounting for the most variance in switching behavior.

For data from the Choices condition, AIC metrics indicated that models incorporating brain activity possessed significantly more explanatory power than that employing only the average probability of switching and the group alignment metric (Figure 4A depicts the results of these tests for each region). This was true for all regions tested, with the insula accounting for the most variance (p<10^−14^ for the model employing only the average probability of switching, p<10^−4^ for the model including group alignment, corrected for multiple comparisons). Similarly, for data from the Rewards condition, brain activity in every region tested provided significantly more explanatory power than the models employing only the average probability of switching and the reward rank metric (Figure 4B), with the insula again accounting for the most variance (p<10^−12^ for the model employing only the average probability of switching, p<10^−4^ for the model including reward rank, corrected for multiple comparisons). Analysis of the data from the Both condition demonstrated that only the insula, thalamus, and dACC provided explanatory power significantly greater than the model using only the average probability of switching and the social information metrics (Figure 4C), with the insula once again accounting for the most variance (p<10^−22^ for the model employing only the average probability of switching, p<.05 for the model including social information, corrected for multiple comparisons). Because the insula was the strongest predictor of switching for each of the social conditions, we further examined the results of the regressions using insula activity to determine the direction and distribution of effects across participants.

**Figure 5:**
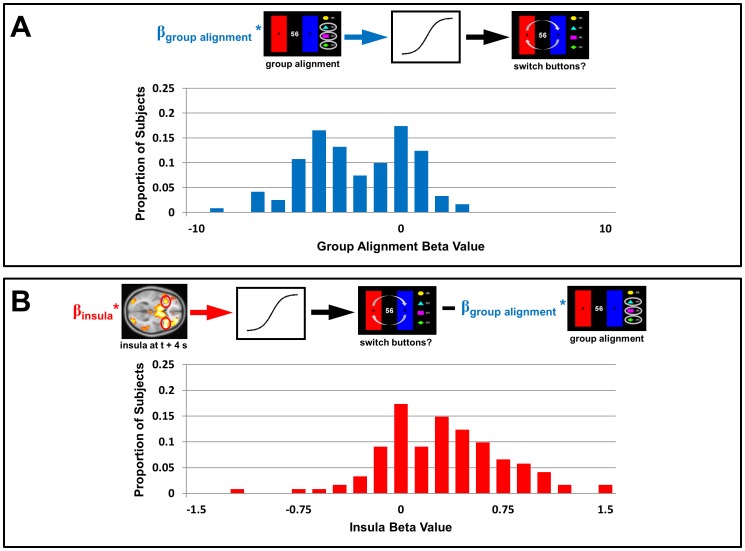
Hierarchical logistic regression of switching behavior in the Choices condition. (A) Behavioral data alone. Using behavioral data from the Choices condition, the group alignment metric was used to predict when participants switched buttons. The beta values (n = 121 values, arbitrary units) for group alignment were significantly less than zero across participants (p<10^−16^), indicating that most participants consistently chose to switch when they were out of alignment with the group. (B) Behavioral data and insula activity. Insula activity for each choice (at time *t+4* seconds, where *t* is the time at which a decision was submitted) was incorporated into the regression model, to test the degree to which this explained switching behavior beyond that explained by group alignment alone. The beta values (n = 121 values, arbitrary units) for insula activity were significantly greater than zero across participants (p<10^−11^), indicating that for most participants increased insula activity was associated with a greater probability of switching, independent of information about group alignment.

We hypothesized that lower values for the social information metrics (group alignment and reward rank) would correspond to higher probabilities of switching; that is, that switching would be more frequent when participants were misaligned with the group, or possessed a low rank in earnings. We further hypothesized that, since insula activity increased with marginality, additional activity in this region would be correlated with higher probabilities of switching. Beginning with data from the Choices condition, the hierarchical logistic regression revealed that the beta values for the effects of social information (Figure 5A) were significantly less than zero (p<10^−16^), indicating that lower values for the group alignment metric (corresponding to greater marginality) were indeed associated with higher probabilities of switching. That is, the less aligned participants' previous choices were with those of other group members, the more likely they were to switch buttons. Conversely, beta values for the effects of insula activity were significantly greater than zero (Figure 5B, p<10^−11^), indicating that greater activity in the insula was associated with higher probabilities of switching.

**Figure 6:**
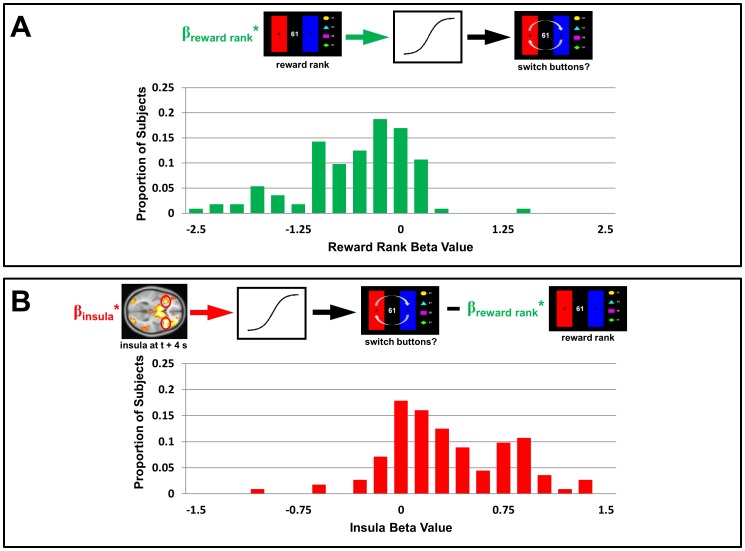
Hierarchical logistic regression of switching behavior in the Rewards condition. (A) Behavioral data alone. Using behavioral data from the Rewards condition, the reward rank metric was used to predict when participants switched buttons. Two participants exhibited beta values more than three standard deviations from the mean beta values, and were excluded (leaving n = 112 values, arbitrary units). The beta values for reward rank were significantly less than zero across participants, indicating the degree of association with switching (p<10^−12^), indicating that lower reward rank was associated with an increased probability of switching. (B) Behavioral data and insula activity. Insula activity for each choice (at time *t+4* seconds, where *t* is the time at which a decision was submitted) was incorporated into the regression model, to test the degree to which this explained switching behavior beyond that explained by reward rank alone. The beta values (n = 112 values, arbitrary units) for insula activity were significantly greater than zero across participants (p<10^−12^), indicating that for most participants increased insula activity was associated with increased probability of switching independent of information about reward rank.

A comparable analysis of the Rewards condition produced similar effects: beta values for reward rank were significantly less than zero (Figure 6A; p<10^−12^), indicating that lower rankings were associated with higher probabilities of switching. Beta values for insula activity were again significantly greater than zero (Figure 6B; p<10^−12^) indicating, as in the Choices condition, that greater insula activity was associated with a greater probability of switching.

**Figure 7:**
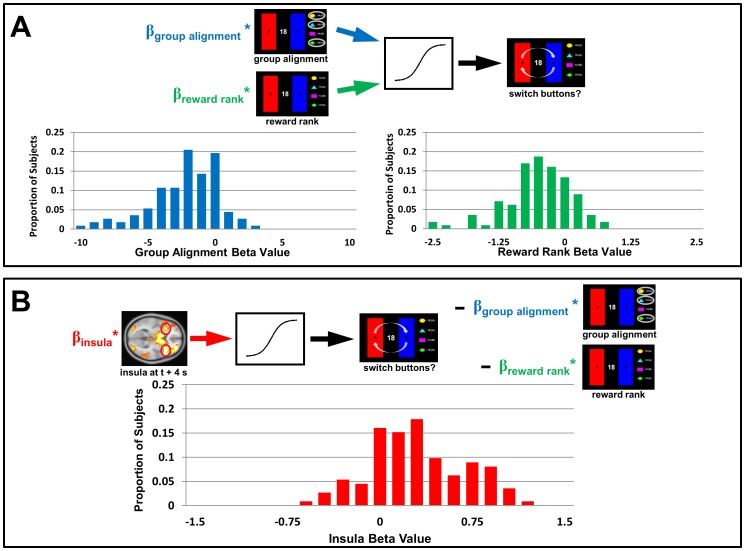
Hierarchical logistic regression of switching behavior in the Both condition. (A) Behavioral data alone. Using behavioral data from the Both condition, group alignment and reward rank were used to predict when participants switched buttons. Three participants exhibited beta values more than three standard deviations from the mean beta values, and were excluded (leaving n = 112 values, arbitrary units). Beta values for group alignment were significantly less than zero across participants (p<10^−15^), indicating that, as in the Choices condition, misalignment with the group was associated with a greater probability of switching. The beta values for reward rank were only marginally different from zero (p = .07). **(B) Behavioral data and insula activity.** Insula activity for each choice (at time *t+4* seconds, where *t* is the time at which a decision was submitted) was incorporated into the regression model, to test the degree to which insula activity explained switching behavior beyond that explained by group alignment and reward rank. The beta values (n = 112 values, arbitrary units) for insula activity were significantly greater than zero across participants (p<10^−12^), indicating that for most participants increased insula activity was associated with a greater probability of switching independent of social information of either type.

Finally, we applied the same analysis to the Both condition by including the group alignment and reward rank metrics in the regression (Figure 7A). Analysis revealed that the beta values for group alignment were significantly less than zero (p<10^−15^), as they had been in the Choices condition. However, beta values for reward rank were only marginally less than zero (p = .07) while BOLD activity in the insula yielded beta values that were once again significantly greater than zero (Figure 7B; p<10^−12^), indicating that switching behavior was influenced more by others' choices and insula activity than by relative earnings when both types of social information were available.

## Discussion

In this study, we used two alternative forced choice tasks that have been used extensively in previous research on decision making to examine the influence of social information and identify the neural systems that mediate this influence. The design of the experiment allowed us to generate two types of veridical yet quantifiable social information on a trial-by-trial basis, to control the type(s) of information to which participants had access during decision making behavior, and to measure the extent to which this information influenced participants' decisions to align themselves with the group. We found that social information was correlated with neural responses in a set of brain regions that showed greater activity when participants were misaligned with the group. Importantly, activity in these regions – and most notably, the insula – predicted subsequent behavior above and beyond social information alone. Our results suggest that the insula and, to a lesser extent, other brain areas (including portions of the dACC and basal ganglia) play an important role in detecting when an individual's behavior differs from that of other group members, and in initiating behavior that realigns the individual with the group.

Previous studies have examined the neural correlates of responding to a social partner's behavior, and found responses in a similar set of brain regions. In an imaging study employing the “ultimatum game,” the DLPFC and insula responded to unfair monetary offers made by a social partner 18, with insula activity differing according to the acceptance or rejection of the offer. Our results parallel these: the DLPFC and insula were both among the regions responsive to misalignment with the group, and the insula was the region most strongly associated with the behavioral response to this social standing. Research conducted using another two-person economic exchange task revealed that insula activity increased as participants were trusted with less money by social partners, and as participants themselves gave less money in return 17. Again, responses in the insula were sensitive to social information indicating deviation from or subordination within the interaction. However, there are two important differences between these studies and the work presented here.

First, because group members' earnings in our tasks were independent and insula activity was independent of *absolute* reward, our findings additionally show that the social phenomena to which the insula responds can be independent of primary reward gained by the participant. Second, the roles in these prior experiments were asymmetric – the actions available to one participant were not the same as those available to the other, and therefore prevented the possibility of realigning to an observed behavior. The combination of these facts with the smaller number of decisions experienced by participants (ten in each of the aforementioned studies, compared with 150 per task in our experiment) may help to explain why our paradigm revealed additional regions that were parametrically sensitive to components of social influence.

Prior research has also investigated the neural correlates of group influences on decision making. In one study by Berns et al. 19, participants made perceptual judgments while a group of confederates gave answers that were often incorrect. Analysis revealed that the decision to conform to the majority was associated with elevated BOLD responses in parietal cortex. This effect is paralleled by the activity of the parietal cortex in our study (see Figure 2), although this region did exhibit as strong an effect as the insula. Berns et al. interpreted their findings as evidence that social information biased computation in the parietal cortex, which was already strongly activated by the perceptual judgments. Our tasks did not require perceptual discriminations, which may explain the diminished involvement of parietal cortex.

In another experiment, participants rated the attractiveness of a series of faces, with each choice followed by the rating from a group of participants 10. In this study, deviations from the group's judgment led to elevated BOLD responses in the dACC; we similarly observed BOLD responses in this region that were sensitive to negative social standing in a graded fashion. This effect is similar to that observed by Burke et al. 14, who found that ACC activity was elevated when participants made decisions that contravened those recommended by a human partner. Klucharev et al. also found significant insula activation in response to deviations from the group's judgment, a finding paralleled by another study which found that the anterior insula was activated when participants contravened advice given to them during an economic decision making task 16. Two additional studies showed that activity in the insula exhibited an interaction between the amplitude of a social judgment and the susceptibility of participants' behavior to social influence 8,9.

Our findings are also consistent with those of other previous experiments investigating the effects of changes in social standing during multi-person interactions. One study examining the neural correlates of social rejection 6 showed that the dACC was more active when participants were excluded from a group. In another study that examined the effects of potential losses of social standing, participants were placed in a group with two other individuals and ranked based on their ability to make correct perceptual judgments 20. Both the insula and dACC exhibited greater activity when participants performed worse than group members previously deemed to be less skilled. Finally, a third study demonstrated the sensitivity of the dACC to social judgments coming from peers, although this activity was not sensitive to the valence of these judgments 7. Our findings parallel these observations, indicating that such responses occur even when social judgments, social hierarchies, or ingroup/outgroup distinctions are not explicit.

Taken together, these findings provide strong evidence in support of a network of brain areas – including insula and dACC – that are responsive to information about social standing. However, in these previous studies, neural activity was observed as a consequence, or correlate of social information. Our findings go beyond this, to show that neural activity in these regions can predict subsequent decision making, consistent with – but also above and beyond – the influence of social information. This strengthens the idea that these regions mediate the influence of social information on behavior that promotes social alignment.

Previous work has also examined the insula using non-social tasks, and has suggested a role for the insula in action selection, as opposed to outcome processing 35. The association between insula activity and switching behavior suggested by our findings provides quantitative support for this claim, demonstrating that insula activity predicted behavioral outcome above and beyond the effects of social information.

The simple, two alternative forced choice decision making tasks used in our study were chosen in part because they are amenable to (and have previously been subjected to) computational modeling, with the goal of extending these models to address the influence of social information on decision making. Ongoing work has begun to address this goal, revealing ways in which social information can be incorporated into simple reinforcement learning models to predict behavior 21–23. An important goal in future work will be to integrate such computational efforts with neuroimaging findings of the sort reported here, to better understand the computational functions subserved by the neural mechanisms that mediate the influence of social information on decision making behavior.

## Supporting Information

Text S1 **Supporting methods.** Additional details are given regarding the structure of the individual decision-making tasks, as well as the organization of the data used in the hierarchical logistic regression. (DOCX)

Figure S1 **Reward functions for the multi-person decision-making tasks.** For a single trial, reward was determined by two variables: (1) whether button “A” (red line) or “B” (blue line) was most recently pressed, and (2) the percentage of the last twenty choices allocated to button “A” (X axis, 0% to 100%, plotted in increments of 5%). The dotted black line depicts the average reward received for each %A. Two of the six tasks were mirrored versions of the “simple rising optimum” and “complex rising optimum” tasks, and are not shown. (TIF)

## References

[1] Egelman DM, Person C, Montague PR (1998) A computational role for dopamine delivery in human decision-making. J Cogn Neurosci 10: 623–630. 9802995 10.1162/089892998563022

[2] Bogacz R, Brown ET, Moehlis J, Hu P, Holmes P, et al (2006) The physics of optimal decision making: A formal analysis of models of performance in two-alternative forced choice tasks. Psychological Review 113: 700–765. 17014301 10.1037/0033-295X.113.4.700

[3] Bogacz R, McClure SM, Li J, Cohen JD, Montague PR (2007) Short-term memory traces for action bias in human reinforcement learning. Brain Research 1153: 111–121. 17459346 10.1016/j.brainres.2007.03.057

[4] Montague PR, Dayan P, Sejnowski PD (1996) A framework for mesencephalic dopamine systems based on predictive Hebbian learning. J Neurosci 16: 1936–1947. 8774460 10.1523/JNEUROSCI.16-05-01936.1996 PMC6578666

[5] Sutton RS, Barto AG (1998) Reinforcement learning: an introduction. Cambridge, MA: MIT Press. 322 p.

[6] Eisenberger NI, Lieberman MD, Williams KD (2003) Does rejection hurt? An fMRI study of social exclusion. Science 302: 290–292. 14551436 10.1126/science.1089134

[7] Izuma K, Saito DN, Sadato N (2008) Processing of social and monetary rewards in the human striatum. Neuron 58: 284–294. 18439412 10.1016/j.neuron.2008.03.020

[8] Berns GS, Capra CM, Moore S, Noussair C (2010) Neural mechanisms of the influence of popularity on adolescent ratings of music. NeuroImage 49: 2687–2696. 19879365 10.1016/j.neuroimage.2009.10.070 PMC2818406

[9] Campbell-Meiklejohn DK, Bach DR, Roepstorff A, Dolan RJ, Frith CD (2010) How the opinion of others affects our valuation of objects. Curr Biol 20: 1165–1170. 20619815 10.1016/j.cub.2010.04.055 PMC2908235

[10] Klucharev V, Hytönen K, Rijpkema M, Smidts A, Fernández G (2009) Reinforcement learning signal predicts social conformity. Neuron 61: 140–151. 19146819 10.1016/j.neuron.2008.11.027

[11] Mason MF, Dyer R, Norton MI (2009) Neural mechanisms of social influence. Organ Behav Hum Dec 110: 152–159.

[12] Zaki J, Schirmer J, Mitchell JP (2012) Social influence modulates the neural computation of value. Psychol Sci 22: 894–900. 10.1177/0956797611411057 21653908

[13] Behrens TEJ, Hunt LT, Woolrich MW, Rushworth MFS (2008) Associative learning of social value. Nature 456: 245–250. 19005555 10.1038/nature07538 PMC2605577

[14] Burke CJ, Tobler PN, Schultz W, Baddeley M (2010) Striatal BOLD response reflects the impact of herd information on financial decisions. Front Hum Neurosci 4: 1–11. 20589242 10.3389/fnhum.2010.00048 PMC2892997

[15] Delgado M, Frank RH, Phelps EA (2005) Perceptions of moral character modulate the neural systems of reward in the trust game. Nat Neurosci 8: 1611–1618. 16222226 10.1038/nn1575

[16] Engelmann JB, Capra CM, Noussair C, Berns GS (2009) Expert financial advice neurobiologically “offloads” financial decision-making under risk. PLoS One 4: e4957. 19308261 10.1371/journal.pone.0004957 PMC2655712

[17] King-Casas B, Sharp C, Lomax-Bream L, Lohrenz T, Fonagy P, et al (2008) The rupture and repair of cooperation in borderline personality disorder. Science 321: 806–810. 18687957 10.1126/science.1156902 PMC4105006

[18] Sanfey AG, Rilling JK, Aronson JA, Nystrom LE, Cohen JD (2003) The neural basis of economic decision-making in the ultimatum game. Science 300: 1755–1758. 12805551 10.1126/science.1082976

[19] Berns GS, Chappelow J, Zink CF, Pagnoni G, Martin-Skurski MEM, et al (2005) Neurobiological correlates of social conformity and independence during mental rotation. Biol Psychiatry 58: 245–253. 15978553 10.1016/j.biopsych.2005.04.012

[20] Zink CF, Tong Y, Chen Q, Bassett DS, Stein JL (2008) Know your place: neural processing of social hierarchy in humans. Neuron 58: 273–283. 18439411 10.1016/j.neuron.2008.01.025 PMC2430590

[21] Nedic A, Tomlin D, Holmes P, Prentice DA, Cohen JD (2012) A decision task in a social context: human experiments, models, and analyses of behavioral data. Proc IEEE 100: 713–733.

[22] Stewart A, Cao M, Nedic A, Tomlin D, Leonard N (2012) Towards human-robot teams: model-based analysis of human decision making in two-alternative choice tasks with social feedback. Proc IEEE 100: 751–775.

[23] Woodruff C, Vu L, Morgansen KA, Tomlin D (2012) Deterministic modeling and evaluation of decision-making dynamics in sequential two-alternative forced choice tasks. Proc IEEE 100: 734–750.

[24] Montague PR, Berns GS, Cohen JD, McClure SM, Pagnoni G, et al (2002) Hyperscanning: simultaneous fMRI during linked social interactions. Neuroimage 16: 1159–1164. 12202103 10.1006/nimg.2002.1150

[25] Li J, McClure SM, King-Casas B, Montague PR (2006) Policy adjustment in a dynamic economic game. PLoS One 1: e103. 17183636 10.1371/journal.pone.0000103 PMC1762366

[26] Kwong KK, Belliveau JW, Chesler DA, Goldberg IE, Weisskoff RM, et al (1992) Dynamic magnetic resonance imaging of human brain activity during primary sensory stimulation. Proc Natl Acad Sci U S A 89: 5675–5679. 1608978 10.1073/pnas.89.12.5675 PMC49355

[27] Ogawa S, Lee TM, Nayak AS, Glynn P (1990) Oxygenation-sensitive contrast in magnetic resonance image of rodent brain at high magnetic fields. Magn Reson Med 1: 68–78. 10.1002/mrm.1910140108 2161986

[28] Frackowiak RSJ, Friston KJ, Frith CD, Dolan RJ, Mazziotta JC (1997) Human brain function. San Diego, CA: Academic Press.

[29] SPM website. Available: http://www.fil.ion.ucl.ac.uk/spm. Accessed 2012 Nov 28.

[30] Friston KJ, Holmes AP, Poline J-B, Grasby PJ, Williams SCR, et al (1995) Analysis of fMRI time-series revisited. Neuroimage 2: 45–53. 9343589 10.1006/nimg.1995.1007

[31] FSL website. Available: http://fsl.fmrib.ox.ac.uk/fsl/fslwiki/. Accessed 2012 Nov 28.

[32] Nichols TE, Holmes AP (2001) Nonparametric permutation tests for function neuroimaging: a primer with examples. Hum Brain Mapp 15: 1–25. 10.1002/hbm.1058 PMC6871862 11747097

[33] Smith SM, Nichols TE (2009) Threshold-free cluster enhancement: addressing problems of smoothing, threshold dependence and localization in cluster inference. NeuroImage 44: 83–98. 18501637 10.1016/j.neuroimage.2008.03.061

[34] Mathworks website. Available: http://www.mathworks.com. Accessed 2012 Nov 28.

[35] Paulus MP, Feinstein JS, Leland D, Simmons AN (2005) Superior temporal gyrus and insula provide response and outcome-dependent information during assessment and action selection in a decision-making situation. Neuroimage 25: 607–615. 15784440 10.1016/j.neuroimage.2004.12.055

[36] NEMO website. Available: http://labs.vtc.vt.edu/hnl/nemo/index.html. Accessed 2012 Nov 28.

