# Supplementary material for: The Neural Substrates of Social Influence on Decision Making
Source: PLoS One. 2013 Jan 9;8(1):e52630. doi: 10.1371/journal.pone.0052630 (PMC3541381; doi:10.1371/journal.pone.0052630)
Supplement: Text S1 — (DOCX) [file pone.0052630.s001.docx]

**Supporting Text S1**

**The Neural Substrates of Social Influence on Decision Making**

Damon Tomlin^1^, Andrea Nedic^2^, Deborah A. Prentice^3^, Philip Holmes^1, 4, 5^, and Jonathan D. Cohen^1, 3^

1. Princeton Neuroscience Institute, Princeton University, Princeton, New Jersey, USA
2. Department of Electrical Engineering, Princeton University, Princeton, New Jersey, USA
3. Department of Psychology, Princeton University, Princeton, New Jersey, USA
4. Department of Mechanical and Aerospace Engineering, Princeton University, Princeton, New Jersey, USA
5. Program in Applied and Computational Mathematics, Princeton University, Princeton, New Jersey, USA

Corresponding Author:

Damon Tomlin

3-N-12 Green Hall

Princeton Neuroscience Institute

Princeton, NJ 08540

Tel: (609) 258 7511

[dtomlin@princeton.edu](mailto:dtomlin@princeton.edu)

**Description of Individual Decision-Making Tasks**

As mentioned in the main text, the experiment consisted of six two alternative forced choice tasks. The reward for a single trial in a task was a function of two variables: which of the two buttons (“A” or “B”) the participant had most recently pressed, and the fraction of the last twenty choices allocated to button “A.” The functional forms of the tasks are shown in Figure S1. Two of the tasks, based on the “rising optimum” task [1], were designed to elicit suboptimal behavior. They featured a local reward maximum separated from a global maximum by a span of low reward; choice histories were initialized to the local maximum, thereby forcing participants to endure a period of low reward in order to discover the global maximum. The functions’ curvature also encouraged adherence to the local maximum. The additional designations of “simple” and “complex” describe the complexity of the strategy required to remain at the global optimum: doing so for the “simple rising optimum” task required only that the participant select button “A” for every choice, while the “complex rising optimum” task required participants to maintain an allocation of 75% A.

Two more tasks were simply “mirrored” versions of these first two: while the first two tasks shown in Figure S1 had global optima at 100% and 75% A, the “mirrored” versions had optima at 0% and 25% A (these are not depicted in Figure S1). Rewards in the two remaining tasks were determined by Gaussian functions, and both had unique global maxima at 50% A. The first of these (“Diverging Gaussians”) possessed an unstable equilibrium which pushed participants equally to either side of the maximum, while the second (“Converging Gaussians”) produced rapid convergence on the maximum. The order of the tasks was randomized across groups, and the crossing of tasks and social conditions (a 6 x 4 design) was balanced across groups.

**Behavioral Modeling**

As groups experienced different combinations of social conditions and tasks (in order to balance the combinations of these manipulations across the data), some individuals experienced the Choices condition twice (contributing two beta values), while the remaining participants contributed only one. Data from the Rewards condition were similary structured, with each participant providing either one or two beta values. The hierarchical logistic regression consequently produced sets of 121 beta values (one set per parameter in the model) for the Choices condition, and 112 sets for both the Rewards and Both conditions.

Although the various tasks elicited different behaviors from the participants, especially when comparing the rising optimum and Gaussian tasks, the results of the hierarchical regression were significant even when the analyses were performed separately for the two classes of reward functions.

**References**

1. Egelman DM, Person C, Montague PR (1998) A computational role for dopamine delivery in human decision-making. J Cogn Neurosci 10: 623-630
