# Supplementary figures and images for: The Neural Substrates of Social Influence on Decision Making
Source: PLoS One. 2013 Jan 9;8(1):e52630. doi: 10.1371/journal.pone.0052630 (PMC3541381; doi:10.1371/journal.pone.0052630)

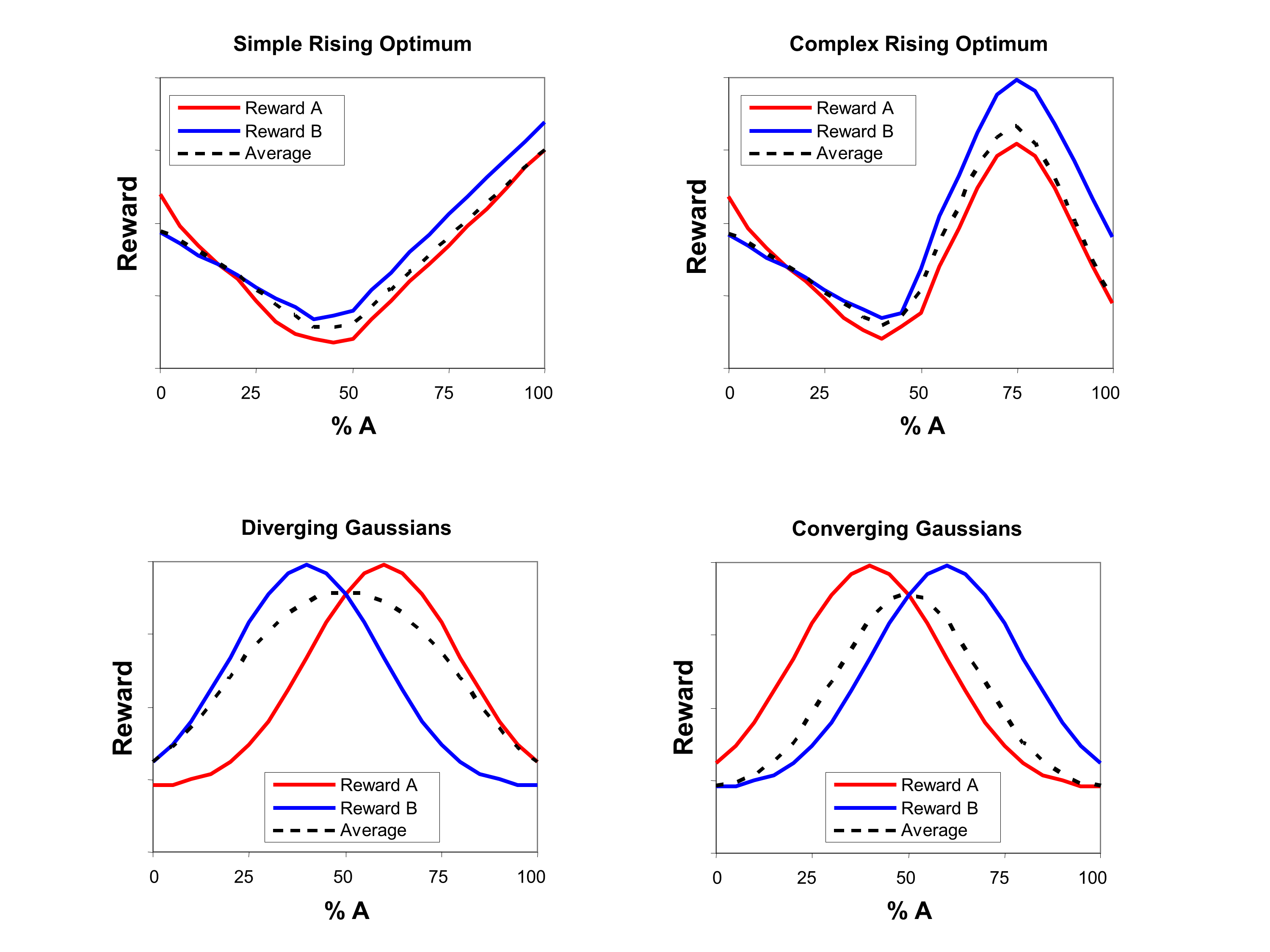

Supplement: Figure S1 — (TIF) [file pone.0052630.s002.tif]
